# Hypoactivation in right inferior frontal cortex is specifically associated with motor response inhibition in adult ADHD

**DOI:** 10.1002/hbm.22539

**Published:** 2014-05-13

**Authors:** Sharon Morein‐Zamir, Chris Dodds, Tim J. van Hartevelt, Wolfgang Schwarzkopf, Barbara Sahakian, Ulrich Müller, Trevor Robbins

**Affiliations:** ^1^ Behavioural and Clinical Neuroscience Institute University of Cambridge Cambridge United Kingdom; ^2^ Department of Psychiatry University of Cambridge Cambridge United Kingdom; ^3^ Department of Psychology University of Cambridge Cambridge United Kingdom; ^4^ Department of Psychology University of Exeter Devon United Kingdom; ^5^ Department of Psychiatry University of Oxford Oxford United Kingdom; ^6^ Department of Psychology Ludwig‐Maximilians‐University Germany; ^7^ Adult ADHD Service Cambridgeshire & Peterborough NHS Foundation Trust

**Keywords:** attention deficit disorder with hyperactivity/diagnosis, magnetic resonance imaging/methods, prefrontal cortex/physiopathology, attention, inhibition control, cognitive flexibility, executive‐function

## Abstract

Adult ADHD has been linked to impaired motor response inhibition and reduced associated activation in the right inferior frontal cortex (IFC). However, it is unclear whether abnormal inferior frontal activation in adult ADHD is specifically related to a response inhibition deficit or reflects a more general deficit in attentional processing. Using functional magnetic resonance imaging, we tested a group of 19 ADHD patients with no comorbidities and a group of 19 healthy control volunteers on a modified go/no‐go task that has been shown previously to distinguish between cortical responses related to response inhibition and attentional shifting. Relative to the healthy controls, ADHD patients showed increased commission errors and reduced activation in inferior frontal cortex during response inhibition. Crucially, this reduced activation was observed when controlling for attentional processing, suggesting that hypoactivation in right IFC in ADHD is specifically related to impaired response inhibition. The results are consistent with the notion of a selective neurocognitive deficit in response inhibition in adult ADHD associated with abnormal functional activation in the prefrontal cortex, whilst ruling out likely group differences in attentional orienting, arousal and motivation. *Hum Brain Mapp 35:5141–5152, 2014*. © **2014 The Authors. Human Brain Mapping Published by Wiley Periodicals, Inc.**

## INTRODUCTION

Attention deficit hyperactivity disorder (ADHD) is characterized by symptoms of inattention, hyperactivity and impulsivity [American‐Psychiatric‐Association, 2000]. Although much focus has been on childhood ADHD, it is evident that symptoms frequently continue into adulthood [Biederman, 2005; Faraone et al., 2000; Wender et al., 2001]. **S**ignificant persistent functional impairments and changes in clinical phenomenology along with maturational changes in cognition, brain structure and function advocate the importance of examining adult ADHD [Biederman and Faraone, 2002; Faraone et al., 2000]. In adults, cognitive impairments as well as functional abnormalities and to a lesser degree subtle structural alterations have been reported [Cubillo et al., 2012; Hervey et al., 2004; Seidman et al., 2005]. As in child ADHD, abnormalities in adults appear to encompass the pre‐frontal cortex (PFC) and its connections to the striatum with additional networks involving the cerebellum and parietal cortex [Castellanos and Proal, 2012; Cortese et al., 2012; Cubillo et al., 2012; Hart et al., 2013].

Prominent psychological theories have implicated inhibitory control in ADHD symptoms, potentially mediating difficulties in impulsivity [Barkley, 1997; Quay, 1997]. Inhibitory control of actions, also termed motor or response inhibition, is the suppression of inadequate, prepotent or ongoing response tendencies [Barkley, 1997; Nigg, 2001], and is considered distinct from but related to other executive functions, such as shifting and information updating [Miyake et al., 2000]. Together these comprise a collection of interrelated cognitive processes enabling flexible, goal‐directed behaviors [Pennington and Ozonoff 1996; Stuss and Alexander, 2000]. Inhibitory dysfunction in ADHD, particularly in response inhibition as measured by go/no‐go and stop signal tasks, has been observed, in combination with atypical activation in frontostriatal and frontoparietal regions [Dickstein et al., 2006; Hart et al., 2013; Lijffijt et al., 2005; Willcutt et al., 2005].

The presence of deficits in other cognitive functions in ADHD has led some to question the relative importance of response inhibition difficulties in ADHD [Castellanos et al., 2006; Willcutt et al., 2005]. In children, meta‐analyses have indicated that effect sizes for response inhibition impairments are similar in magnitude to those found for other executive functions, such as spatial planning [Nigg, 2005; Willcutt et al., 2005]. Additionally, the neural substrates mediating response inhibition overlap substantially with those underlying other executive functions [Derrfuss et al., 2005], where similar abnormalities in ADHD have been found [Cortese et al., 2012; Dickstein et al., 2006]. Moreover, a meta‐analysis of the stop signal task indicated that longer response inhibition latencies in children with ADHD were proportional compared with their general slowing [Lijffijt et al., 2005]. The authors concluded that the behavioral impairments of these children were not specific to deficient inhibitory control but could reflect additional abnormal processes for example, relating to attention [Lijffijt et al., 2005].

While there has been considerably less research into adult ADHD, the behavioral evidence for inhibitory deficits appears robust, with some support for its specificity compared with other functions [Bekker et al., 2005; Boonstra et al., 2010]. In fact, the above stop signal meta‐analysis provided support for a specific response inhibition deficit in adults [Lijffijt et al., 2005]. However, as in children, there is also evidence for widespread executive impairments [Biederman et al., 2011; Boonstra et al., 2005; Hervey et al., 2004; McLean et al., 2004] consistent with the notion that inhibitory deficits may exist as part of a more general pattern of executive dysfunction [Nigg, 2001]. Similarly, neuroimaging evidence in adult ADHD suggests functional abnormalities in a range of executive functions including response inhibition, working memory and task switching [Cortese et al., 2012; Cubillo et al., 2012]. However results are mixed, with adult patients showing reduced, increased or no difference in activation in fronto‐striatal substrates during task performance [Carmona et al., 2012; Cubillo et al., 2010; Dibbets et al., 2010, 2009; Dillo et al., 2010; Epstein et al., 2007; Kooistra et al., 2010; Mulligan et al., 2011]. Moreover, varied results whilst comparing control and patient groups have been noted in the inferior frontal cortex (IFC) [Carmona et al., 2012]. Additional inconsistent results have been reported in the cerebellum and parietal cortex [Dillo et al., 2010; Epstein et al., 2009; Hale et al., 2007].

An important source of variability in the findings comes from differences in task demands. These are exacerbated even further by differences compared with healthy controls in patients' levels of sustained attention, motivation and arousal, which also likely fluctuate between studies. All three factors are believed to play a pivotal role in ADHD [Castellanos et al., 2006; Sergeant, 2005]. This is especially important when examining response inhibition in commonly used go/no‐go and stop signal tasks, as relatively infrequent stop or no‐go stimuli are typically compared with frequent go trials. This confounds attentional processing demands, such as attentional orienting and target detection with response control and inhibition [Dodds et al., 2011; Levy and Wagner, 2011] although see [Smith et al., 2006] for an exception). As attentional orienting and response control and inhibition activate overlapping fronto‐parietal regions [Dodds et al., 2011; Hampshire et al., 2010; Sharp et al., 2010], it may well be that group differences in motor‐inhibition reported in recent meta‐analyses [Cortese et al., 2012; Hart et al., 2013] may not be attributable to response‐inhibition or response‐control abnormalities per se.

In the present study we directly compared response inhibition and attentional shifting between adult ADHD with no comorbidities and matched healthy controls, to examine the specificity of response inhibition deficits. In healthy adults, this task has previously revealed greater activation in the right IFC during inhibition relative to shifting and greater activation in the left inferior parietal cortex (IPC) during shifting relative to inhibition [Dodds et al., 2011].This dissociation was observed alongside extensive co‐activation during both response inhibition and shifting in frontoparietal regions including the IFC. The right IFC appears to play an important role in response inhibition and response control in general [Aron et al., 2003; Dodds et al., 2011], but as noted above is also associated with multiple task demands such as attention, orienting and switching [Levy and Wagner, 2011; Wager et al., 2004]. Moreover, reduced activation in adult ADHD in the IFC to date has been noted in a variety of executive tasks including response inhibition and task switching [Cubillo et al., 2010; Mulligan et al., 2011]. We thus sought to examine potential group differences in this region associated specifically with response inhibition rather than more general aspects of executive functions, whilst controlling for general attentional and motivational differences.

## MATERIALS AND METHODS

### Participants

Nineteen adults diagnosed with ADHD were individually matched for age and gender with 19 healthy controls (see Table 1). Control participants were recruited via posters in the local community and from the Behavioural and Clinical Neuroscience Institute participant panel. The data from 15 controls were included in the previous report [Dodds et al., 2011]. Participants with ADHD were recruited from a specialist Adult ADHD Research Clinic in Cambridge. Detailed information regarding diagnostic procedures is provided elsewhere [Chamberlain et al., 2007]. In brief, a diagnosis of ADHD according to DSM‐IV‐TR criteria was contingent upon six (or more) of nine DSM‐IV inattention and/or hyperactivity/impulsivity criteria being met during childhood and during the previous 6 months [American Psychiatric Association, 2000]. Patients undertook an extensive clinical assessment by a psychiatrist specialized in the assessment and treatment of adult ADHD (UM) who determined whether ADHD symptoms interfered significantly with everyday function and were not due to another disorder, together with symptom ratings from the patient, from an informant who had known the patient in childhood (usually a parent), and from an informant who had known the patient during the previous 6 months of adult life [Barkley, 2006; Kessler et al., 2005] see also Weiss Functional Impairment Rating Scale). There were 11 ADHD patients with combined type, six with inattentive type, and two predominantly hyperactive‐impulsive. Nine patients were unmedicated (two were previously medicated), nine were prescribed methylphenidate, and one was prescribed atomoxetine. A confirmed diagnosis of adhd in childhood was available in nine patients (seven currently medicated) though as required, for all patients the extended clinical interview in addition to parent and patient ratings and report of childhood symptoms clearly indicated adhd in childhood. The patients did not satisfy DSM‐IV criteria for any other disorders and exclusion criteria included prior diagnosis of schizophrenia, psychotic disorders, or bipolar disorder; in addition to major depressive disorder, Obsessive Compulsive Disorder or substance abuse in the last 3 months. Over 100 patients were screened over the duration of 18 months to ensure these criteria. For healthy controls, exclusion criteria included no current or past psychiatric disorders and no psychoactive medications. For all participants further exclusion criteria were current or past neurological disorders (including tic disorders), brain damage or MRI contraindications.

**Table 1:** Demographic and clinical characteristics of ADHD and control groups

	Controls		ADHD patients		
Male: female	13:6		13:6		
	Mean	SD	Mean	SD	*P*
Age (yr)	28.579	7.034	29.106	7.716	0.827
Verbal IQ	116.032	8.158	115.650	9.468	0.896
MADRS	5.053	3.704	9.789	7.525	0.018
ASRS	24.895	6.691	48.526	11.801	0.000
CAARS			49.333	8.905	

ADHD: attention deficit/hyperactivity disorder; IQ, intelligence quotient; MADRS, Montromery‐Asberg Depression Rating scale; ASRS: Adult ADHD Self‐Report Sclae; CAARS: Conners' Adult ADHD Rating Scale.

The study was approved by the **l**ocal Research Ethics Committee (08/H0308/65) and participants provided informed consent before study onset and were reimbursed for their participation. To minimize the impact of psychotropic medication, medicated patients discontinued their medication at least 24 h before scanning [Gualtieri et al., 1982]. ADHD symptom severity at testing was assessed using the Connors adult ADHD rating scale short version self‐report (CAARS) [Conners et al., 1999; Erhardt et al., 1999]. All participants completed the Adult ADHD Self‐Report Scale (ASRS) [Kessler et al., 2005], the National Adult Reading Test (NART) [Nelson, 1982] to assess verbal IQ and the Montgomery‐Asberg Depression Rating Scale (MADRS) [Montgomery and Asberg, 1979] to assess depressive symptom severity.

### Procedure

Participants performed a go/no‐go task, described in detail elsewhere [Dodds et al., 2011]. On each trial they were presented with an image of a superimposed face and house. The border color surrounding the image determined the relevant stimulus dimension. In simple blocks the color remained constant and subjects attended to one stimulus dimension (faces or houses) throughout. In complex blocks, every few trials the border color changed, constituting a shift trial, where subjects had to shift attention between stimulus dimensions. The type of response (Go/no‐go) was determined by the gender of the face or the number of storeys of the house. Thus for example, participants were instructed that when the border was blue they must attend to the faces and respond when the face is male and to withhold responding when the face is female. When the boarder was red, they must attend to houses and respond to two‐storey houses but not to one‐storey houses. Participants completed a simple and complex block in each of two runs, with go/no‐go rules and block order counterbalanced across subjects within each group.

On each trial, a red or blue border appeared centrally for 1,000 msec informing participants whether to attend to faces or houses. The image was then presented within the border for 725 msec. On go trials, participants had to respond within this duration using a customized button box resting on their stomach, whereupon the display disappeared. On no‐go, subsequently called stop trials, participants had to refrain from responding. Following a correct response, a blank screen was presented for 1,000 msec, and following an incorrect response negative verbal feedback was presented for the same duration. Total trial length was therefore 2,725 msec, with trial onset jittered relative to the repetition time (TR) of 2,000 msec.

Before scanning, participants practiced both conditions to ensure task and instruction comprehension. Instructions were further presented before each block in the scanner for 10 sec. In the simple version there were a total of 40 stop and 280 go trials, and in the complex version, there were a total of 40 stop, 40 shift, and 240 go trials, yielding the same frequency of stop and shift trials and the same ratio of stop and shift trials to go trials which was approximately 1:7. Blocks, lasting approximately 8.5 min, consisted of approximately 160 trials, with 4 to 12 go trials between consecutive stop trials and between consecutive shift trials.

Stimuli were 80 face‐house pairings with 20 stimuli of each combination of male/female and one‐storey/two‐storey house. The grayscale images were portrayed in a 400 × 400 pixel square with a 5**‐**pixel wide blue or red border. In the complex version, stimuli from the irrelevant dimension were selected equally often. For example, when attending to faces, the irrelevant stimulus was a two**‐**storey house on half the trials and a one‐storey house on the remaining ones. The task was projected onto a mirror in the scanner, and presented via E‐Prime software (Psychological Software Tools, Inc.) on an IBM personal computer running Windows XP.

### Scanning Acquisition

Scanning was carried out at the Wolfson Brain Imaging Centre, Cambridge, on a 3 T Siemens Tim Trio scanner. Functional imaging data were collected in a single session using whole‐brain echo planar images (EPI) with the following parameters: repetition time (TR) = 2,000 msec; echo time (TE) = 30 msec; flip angle = 78°; interleaved sequence; 32 slices with slice thickness 3 mm plus 0.75 mm gap; matrix = 64 × 64; field of view (FOV) = 192 × 192 mm yielding 3 × 3 mm in‐plane resolution; echo spacing 0.47 msec and bandwidth 2,442 Hz/Px. The number of volumes acquired per run varied from 456 to 485 depending on total trial number. Structural T1‐weighted MR scans using a magnetization‐prepared rapid acquisition gradient‐echo (MPRAGE) sequence were used for registration (176 slices of 1 mm thickness, TR = 2,300 msec; TE = 2.98 msec, TI = 900 msec, flip angle = 9°, FOV = 240 × 256 mm).

### Data Analysis

For behavioral data, repeated‐measures analyses of variance (ANOVAs) contrasted group (ADHD vs. controls) on commission errors and on omission errors for each block (simple vs. complex). Additionally, a 2 × 2 × 3 ANOVA compared group correct go reaction times (RT) for face versus house stimuli in simple, complex and switch trials. Where group effects were significant, Cohen's *d* effect sizes were calculated.

Functional magnetic resonance imaging (fMRI) data were processed and analyzed using Statistical Parametric Mapping 8 (SPM8, http://www.fil.ion.ucl.ac.uk/spm/). Images from the first five volumes were discarded to allow for T1 equilibrium effects. Images were slice time corrected and spatially realigned, and then co‐registered to a segmented structural image using the mean functional volume. Normalization to the Montreal Neurological Institute (MNI) template was then followed with resampling of EPI volumes to 2 mm isotropic voxels and smoothing with a 6‐mm full‐width‐half‐maximum Gaussian kernel.

Design‐matrices were implemented using the general linear model (GLM). First level regressors for simple blocks were: correct stop trials and a subset of correct go trials; and for complex blocks: correct stop trials, shift trials, and two subsets of correct go trials. Additional regressors of no interest included incorrect stop trials, and parametric modulators for go and shift RT. Go trials comprised separate random selections from all correct go trials and were matched in number to correct stop or shift trials in that block to control for the same number of trials. As they occurred frequently, they were not expected to be separable from the GLM baseline, but were included to allow for stop and shift contrasts to be entered into subsequent conjunction analyses with separate baselines ([see Dodds et al., 2011] for further details). The regressors, modeled at target onset, were convolved with a canonical hemodynamic response function (HRF). The six realignment parameters and any volumes with excessive movement (greater than 1 voxel, constituting less than 0.5% of volumes) were entered as additional regressors. The data were high‐pass filtered (1/128 Hz cut‐off) to remove low frequency signal drifts and serial correlations were accounted for by a first‐degree autoregressive AR (1) model. Voxel‐wise *t* maps were constructed for each participant for the contrasts of stop vs. shift as well as stop versus go and shift versus go in the complex blocks and simple stop vs. go in the simple blocks. Mean number of trials was 23, 29, and 30 for the complex stop, shift and simple stop contrasts respectively. The resulting contrast images were then used in second level analyses.

Key second level analyses investigated group differences specifically associated with stopping versus shifting. As this contrast targets the difference between overlapping cognitive processes and based on previous findings, we adopted an ROI approach. All participants were entered into one‐sample *t*‐test whole‐brain analyses performed with family‐wise error (FWE) correction set at *P* < 0.05, to probe for brain activations associated exclusively with stopping versus shifting. Group differences were examined using an orthogonal contrast with the resulting functional regions of interest (ROI) constituting 10‐mm spheres, encompassing approximately 523 voxels, surrounding the peak coordinates from the whole‐brain analyses (see [de Wit et al., 2012] for a similar approach). Mean activation was calculated using MarsBar [Brett et al., 2002]. These functional ROIs were also correlated with ADHD severity scores.

Secondary anatomical ROI analyses are also reported, primarily to clarify and describe individual group performance. These encompassed areas hypothesized to be of importance to response inhibition and shifting, such as the inferior frontal gyrus (IFG), anterior insula, pre‐SMA and inferior parietal cortex [Levy and Wagner, 2011; Swick et al., 2011] as well **as** the anterior cingulate (ACC) hypothesized to be of importance to control processes in ADHD [Bush et al., 1999]. These ROIs were taken from the automated anatomical labeling (AAL) atlas [Tzourio‐Mazoyer et al., 2002] with the anterior insula defined from *y* > 0 as was the pre‐SMA, and the IFG comprising the pars opercularis and pars triangularis.

We further investigated activations associated with both stopping and shifting, or with both complex and simple stopping, using second‐level random effects conjunction analyses against the conjunction null hypothesis in whole‐brain analyses. The resulting activations were employed as a search area to inspect for potential group differences. Peak voxels are reported in MNI coordinates. Where appropriate, whole brain family‐wise error corrected analyses are followed up with secondary within ADHD group analyses at uncorrected threshold, *P* < 0.001, to provide a more complete overview of the findings in the ADHD group. This is to counteract concerns that using family wise correction is highly conservative and raises type II error. Moreover, this allows one to avoid any erroneous conclusions regarding lack of activation in the ADHD patients, which may actually be revealed at more liberal thresholds, having important theoretical implications.

## RESULTS

### Demographics and Clinical Measures

The groups were matched for age, gender, and verbal IQ, with ADHD patients reporting increased ADHD symptom severity levels and slightly elevated depression though not in the clinical range (see Table 1).

### Behavioral Measures

ADHD patients made significantly more commission errors than controls: 34.56% versus 23.81%, respectively (*F*_(1,36)_ = 4.95, *P* = 0.032, *D* = 0.72). Although there was an effect of task difficulty (*F*_(1,36)_ = 44.13, *P* < 0.001), with increased commission errors in the complex (35.27%) compared with the simple task (23.09%), there was no group by difficulty interaction (*P* = 0.346). Additionally, ADHD patients had more omission errors on go trials (4.21%) compared with controls (1.67%), (*F*_(1,36)_ = 4.61, *P* = 0.039, *D* = 0.70). There was no effect of difficulty on omission errors, nor was there a group by difficulty interaction (*P* > 0.5).

An ANOVA examined correct go RTs in the two groups in simple, complex no‐shift and shift trials for face and house stimuli. Mean RTs were slower in ADHD versus control participants overall: 610 versus 577 msec, respectively (*F*_(1,36)_ = 4.59, *P* = 0.039, *D* = 0.70), and group did not interact with any other factor (*P* > 0.481). Responses were slower to houses (599 msec) than faces (588 msec) (*F*_(1,36)_ = 16.21**,** *P* < 0.001), but stimuli interacted with task (*F*_(2,72)_ = 6.96, *P* = 0.002) as latencies to houses were slower than faces in the complex task (*F*_(1,36)_ = 37.96, *P* < 0.001) but not in the simple task (*P >* 0.5). Planned comparisons confirmed that latencies to houses were slower than to faces both on complex go trials (*F*_(1,36)_ = 73.18, *P* < 0.001) and shift trials (*F*_(1,36)_ = 15.70, *P* < 0.001), thus, indicating participants successfully shifted their attention on the later trials, with no difference in the magnitude of this effect between complex go and shift trials (*F*_(1,36)_ = 1.15, *P* = 0.291). Importantly, there was also no evidence for slower switching in the ADHD compared with the control group (*F*_(1,36)_ = 0.054, *P* = 0.818).

To adjust for the pre‐existing differences in depression severity and in the case of commission errors also for go RT differences, analyses of covariance were conducted. Group differences in commission errors remained significant when co‐varying depression severity and mean RT, as did group differences for mean RT when co‐varying depression severity. Finally, there were no significant group effects in go SD (*P* > 0.35).

### Neuroimaging

There were no significant group or group by motion effects in the extent of three‐dimensional motion in *x*, *y*, and *z* translations and rotations (*P* > 0.5 for all comparisons).

### Direct Contrast of Stopping Versus Shifting

**Figure 1:**
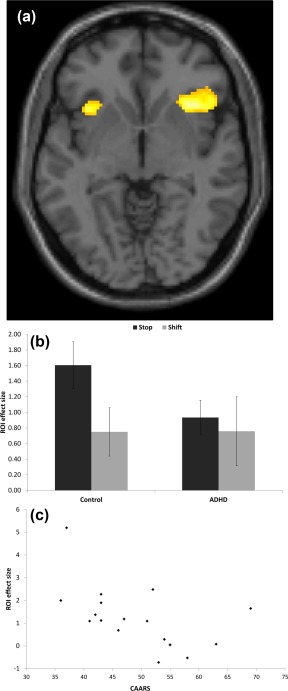
**A**, Greater activation for stopping relative to shifting for all participants, overlaid on the MNI template brain at an uncorrected threshold of *P <* 0.001. **B**, Region of interest activity for stop and shift trials in control and ADHD patients groups in the functional region of interest centered on the MNI peak coordinate = [32, 16, −10] in the right inferior frontal cortex of the contrast stopping relative to shifting. Error bars represent standard error of the mean. **C**, A scatterplot showing negative correlation of Connors adult ADHD rating scale self‐report and region of interest activity in ADHD patients.

The direct contrast of stop minus shift across all participants revealed a single cluster at whole brain FWE corrected level with the peak in the right IFC (*P* = 0.016, peak coordinate = [32, 16, −10], cluster extent (KE) = 5, *Z* = 5.13). The functional ROI analysis revealed a significant group difference in the **IFC** region (*t*_(36)_ = 2.24, *P* =0.03, 1.531 vs. 0.513 for control and ADHD groups contrast values, respectively) as seen in Figure 1. This remained significant when co‐varying for depression levels and go and stop performance. CAARS scores were negatively associated with activation during stopping in this ROI in the ADHD group (*t*_(17)_ = −2.57, *P* = 0.021, *r* = −0.540) and no significant associations with performance were noted. To alleviate concerns of non‐independence, the ROI analysis was repeated using the coordinates derived from a separate sample [Experiment 2, Dodds et al., 2010]. This analysis also revealed a significant group difference for the contrast stop versus shift (*t*_(36)_ = 1.71, *P* = 0.0476, MNI coordinates = [40, 6, 2]).

Inspection of the anatomical ROIs for each group individually revealed in controls greater activation associated with stopping compared with shifting for the right and left anterior insula (*t*_(18)_ = 4.794, *P* < 0.001, mean contrast value = 1.417; and *t*_(18)_ = 3.527, *P* < 0.001, mean contrast value = 0.940, respectively). Similarly greater activation for stopping was observed in the right and left anterior cingulate (*t*_(18)_ = 2.556, *P* = 0.007, mean contrast value = 0.770, and *t*_(18)_ = 2.316, *P* = 0.013, mean contrast value = 0.791, respectively), and marginally greater activation in the right IFG (*t*_(18)_ = 1.61, *P* = 0.057**,** mean contrast value = 0.482). In the ADHD group, there was only marginally greater activation associated with stopping compared with shifting in the anterior right insula (*t*_(18)_ = 1.537, *P* = 0.066, mean contrast value = 0.518). **A** whole brain analysis on the patients for stop versus shift was conducted with an uncorrected threshold *P* < 0.001. This showed a right IFC (peak coordinates = [44, 22, −6], *K*_E_ = 38, *Z* = 3.61), and a small left cluster (peak coordinates = [−34, 18, −6], *K*_E_ = 2, *Z* = 3.23). Although between group comparisons of the anatomical ROIs indicated reduced activation in the ADHD patients in stopping compared with shifting in the anterior insula bilaterally and the anterior cingulate (*P* < 0.05 for all), none of these comparisons survived correction for multiple comparisons.

### Direct Contrast of Shifting Versus Stopping

The contrast of shift minus stop across all participants revealed several activations in the left and right precuneus, and the left cuneus as well as several activations in the cerebellum (see Table 2). Importantly, there were no significant group differences associated with any of the resulting ROIs (*P* > 0.16 for all). We further investigated the activations associated with shifting compared with stopping in the cuneus and precuneus for each group separately as they were not previously noted in healthy adults [Dodds et al., 2011]. Whilst significant activation was associated with shifting versus stopping in both the controls and patients (*P* < 0.001 for all ROIs), significant activation associated with stopping versus go was seen only in controls (*P* < 0.05 for all) but not patients (*P* > 0.19 for all). To complement this, there were significant group differences in stopping relative to go trials in the right precuneus (*t*_(36)_ = 3.23, *P* = 0.001) and left cuneus (*t*_(36)_ = 2.242, *P* = 0.016) ROIs, with a marginal difference in the left precuneus (*t*_(36)_ = 1.322, *P* = 0.09). There were no significant group differences in the shifting relative to go trials in the ROIs and both groups demonstrated significant activation when examined individually (all *P* values <0.002). This, along with inspection of the data, suggests that the difference between shifting and stopping in the cuneus, precuneus and cerebellum resulted from generally reduced activation in complex stopping trials in patients.

**Table 2:** Significant brain regions whilst comparing stopping and shifting in all participants.

	Hemisphere	*Z*‐score	Peak coordinates MNI (mm)			Cluster size (voxel)	Brain region
Contrast			*x*	*y*	*z*		
Stopping > shifting	R	5.15	32	16	−10	5	Anterior insula
Shifting > stopping	R	5.92	18	−60	−20	117	Cerebellum
	R	5.60	12	−66	−46	16	Cerebellum
	R	5.21	20	−48	−22	8	Cerebellum
	R	5.17	10	−64	−14	11	Cerebellum
	R	5.89	4	−68	24	51	Precuneus
	L	5.31	−10	−74	32	9	Cuneus
	L	5.11	−6	−66	50	20	Precuneus

Previously, greater activation associated with shifting compared with stopping was reported in the left inferior parietal cortex [Dodds et al., 2011] though not in posterior medial areas. Inspection of the left inferior parietal cortex ROI revealed significant activation associated with greater shifting compared with stopping in the controls (*t*_(18)_ = 3.309, *P* = 0.001), which was marginal in the patients (*t*_(18)_ = 1.579, *P* = 0.062), however the direct group comparison was not significant (*P* > 0.30).

### Common Activation Associated With Stopping and Shifting

**Figure 2:**
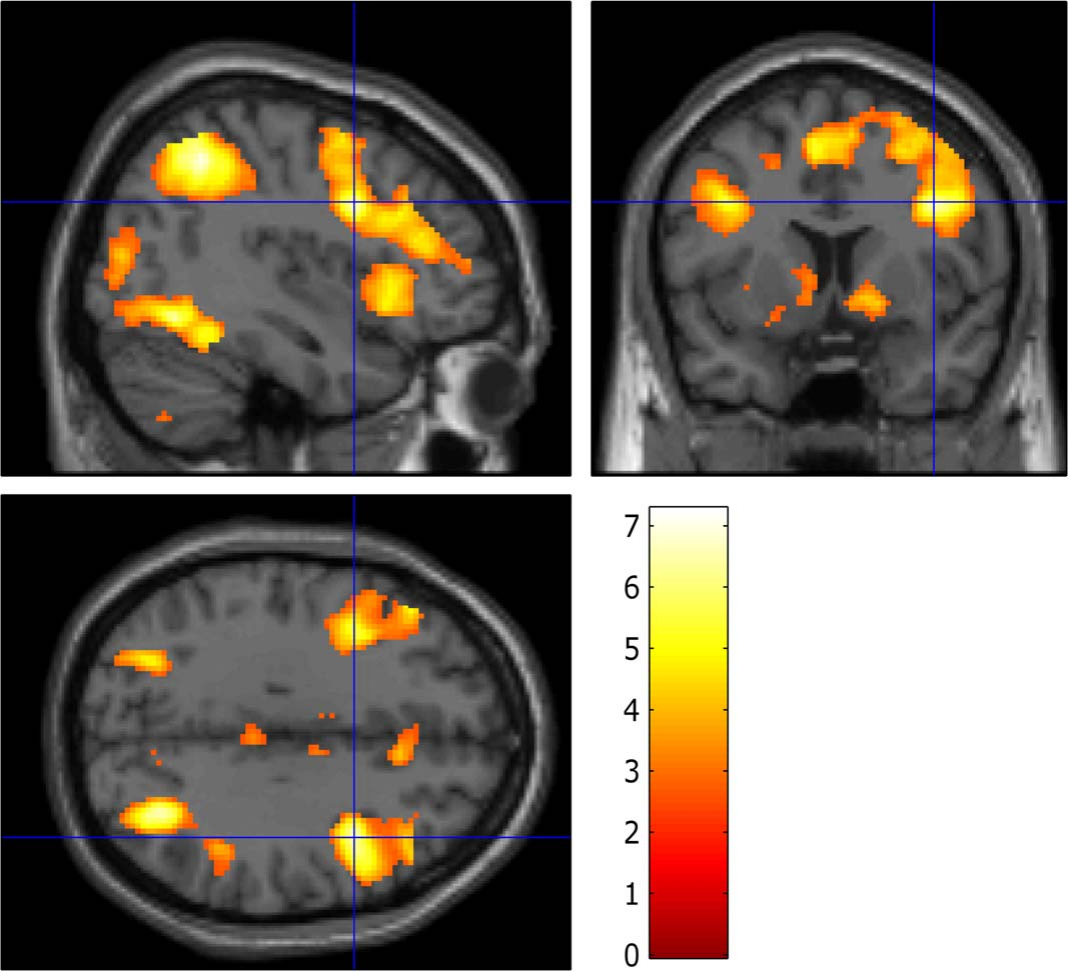
Areas commonly activated during stop and shift trial relative to go trials across all participants overlaid on the MNI brain. Images are displayed at *x* = 40, *y* = 8, and *z* = 32 in the sagittal, coronal, and axial planes, respectively, with a voxelwise threshold of false discover rate *P <* 0.05 for ease of comparison to previous study. Color bars represent *t* scores.

The conjunction analysis across all individuals revealed frontoparietal activations during stop and shift trials compared with go trials at FWE *P* < 0.05 corrected. These areas included clusters in the inferior parietal lobe bilaterally extending into the occipital lobe, as well as anterior clusters extending from the vlPFC to the anterior insula and into the middle frontal gyrus and pre‐SMA bilaterally (see Fig. 2). Activations included the right inferior frontal gyrus (peak coordinate = [40, 8, 32], *K*_E_ = 127) and anterior insula bilaterally (peak coordinates = [34, 22, −4], *K*_E_ = 79 and [−30, 20, 2], *K*_E_ = 74 for right and left, respectively) as well as parieto‐occipital regions of activation (peak coordinates = [32, −66, 42], *K*_E_ = 732 and [−30, 58, 46], *K*_E_ = 274 for right and left, respectively). Similar activations in frontoparietal regions were seen in each group individually, although with reduced extent in the ADHD group. There were no significant group differences in overall average activation in the areas activated in the conjunction analysis, either for stopping (*t*_(36)_ = 0.840, *P* = 0.406) or for shifting (*t*_(36)_ = 0.544, *P* = 0.590). Closer inspection of the ADHD specifically indicated that stopping relative to go trials baseline was associated with activation in the IFC (peak coordinates = [34, 22, −6], *K*_E_ = 10, *Z* = 5.08) FWE *P* < 0.05 corrected. At an uncorrected threshold of *P* < 0.001 activations associated with stopping extended from the right IFG posteriorly into the putamen. Additional activation was noted in right and left parietal and occipital lobes and SMA bilaterally as well as in the left IFC. Shifting in patients was associated with precuneus activation at FWE *P* < 0.05 corrected (peak coordinates = [−2, −0, 46], *K*_E_ = 1, *Z* = 4.83), though at an uncorrected threshold of *P* < 0.001 there were extensive bilateral parietal, precuneus and occipital activations in addition to cerebellar and bilateral IFC and pre‐SMA and striatal activations.

### Stopping in Complex and Simple Blocks

A comparison of complex versus simple stop trials across all participants did not reveal significant differences, although at a more liberal level of *P <* 0.001 uncorrected, bilateral caudate and putamen activations were observed. Simple stop at FWE corrected *P <* 0.05 was associated with bilateral activations in frontoparietal and occipital areas across both groups, particularly in the right IFC, and inferior parietal areas on the right. These regions overlapped to a large degree with those associated with complex stopping and were more pronounced on the right. There were no significant group differences in the average activation in the areas activated in the conjunction analysis, both for complex (*t*_(36)_ = 0.819, *P =* 0.207) and for simple stopping (*t*_(36)_ = 0.451, *P =* 0.673).

As participants performed two stopping tasks, we examined whether there were potential interactions between stopping tasks and groups, as there is evidence of increased activation in ADHD when the task is not difficult. Thus a whole brain conservative analysis investigated whether there were brain regions associated with decreased activation in the ADHD group during the complex stopping condition but increased activation during simple stopping compared with controls. This interaction revealed foci in the left fusiform (peak coordinates = [−30, −64, −6], *K*_E_ = 104, *Z* = 5.02) and right temporal pole (peak coordinates = [46, 6, −12], *K*_E_ = 53, *Z* = 4.99) at FWE *P <* 0.05 corrected, though at *P <* 0.08 there were additional activations in the left superior temporal gyrus (peak coordinates = [−48, −4, −6], *K*_E_ = 90, *Z* = 4.66) and a large activation in the precuneus bilaterally (peak coordinates = [14, −52, 8], *K*_E_ = 1525, *Z* = 4.67). At an uncorrected threshold *P <* 0.001 limited to a twenty voxel cluster extent, extensive activation throughout the precuneus was observed with additional activations in the superior temporal lobes, cerebellum and thalamus. Parallel interaction analyses with shifting did not reveal any significant activations.

### Medication and Gender

Analyses also investigated potential group differences between medicated and unmedicated patients. No significant differences in performance were noted (*P* > 0.3 for all comparisons). Similarly, no differences in functional or anatomical ROIs were noted (*P* > 0.27 for all comparisons). No differences between gender emerged either. When examined separately for males and females, the effect sizes for right IFC ROI hypoactivation in ADHD were highly similar, pointing to medium to large effects (partial eta‐squared values of 0.13 and 0.11 for males and females, respectively).

## DISCUSSION

This study found reduced activation associated with stopping a prepotent action, when controlling for more general attentional and executive control function, in right IFC in adults with ADHD who had no co‐morbidities. Stopping activation was moderately associated with ADHD symptom severity. The present result taken together with the behavioral performance suggest that response inhibition, or response control in general, may be particularly impaired in adults with ADHD [Boonstra et al., 2010; Lijffijt et al., 2005].

Increased fluctuations in motivation or arousal in the ADHD patients or general group differences in these factors [Castellanos et al., 2006; Sergeant 2005] may influence responses to infrequent stop, or no‐go, trials selectively as compared with go trials. However, as stopping was contrasted directly with infrequent shifting, present functional group differences are unlikely to result from such factors. Moreover, this contrast addresses confounds due to task demands associated with the **stop** trials such as attentional orienting, shifting and target detection that would be present also in the healthy controls. The importance of controlling for these processes is highlighted by the fact that IFC activation is observed with such non‐inhibitory executive function processes [Levy and Wagner, 2011]. Moreover, these potential factors, which likely vary between response inhibition studies together with important differences in task demands, contribute to the varied results observed in imaging studies in adult ADHD. Thus, the strength and novelty of the present study is in demonstrating right IFC hypoactivity in ADHD whilst avoiding such potential confounds.

These results reinforce some of the conclusions of recent MRI peak‐coordinate based meta analyses regarding response inhibition abnormalities in adult ADHD [Hart et al., 2013], whilst using a more controlled contrast. Namely, the right IFC was reported as showing decreased activation in adult ADHD patients compared with controls in motor response inhibition tasks where stop or no‐go trials were generally compared with go trials [Hart et al., 2013]. This region has also been highlighted in recent meta‐analyses of motor inhibition tasks in healthy adult populations [Levy and Wagner, 2011; Swick et al., 2011] and is believed to be a node in a large‐scale network involved in cognitive control [Dosenbach et al., 2008]. Moreover, recent evidence suggests that this region of the right IFC is associated with response control and action updating, specifically though not exclusively required in response inhibition [Dodds et al., 2011]. It is particularly important to stress the lack of comorbidities in the present patient sample, as the hypothesis pertained to whether response inhibition specifically is associated with abnormalities in the right IFC in ADHD per se.

Group differences in the ACC and SMA were also noted in the PFC in the recent meta‐analysis as showing reduced activation relating to motor inhibition [Hart et al., 2013; see also Bush et al., 2005]. Our results are in line with ACC but not SMA abnormalities, as being specific to motor inhibition and response control in the present task, though the analysis did not survive correction for multiple comparisons. Interestingly, SMA abnormalities may be ameliorated in adult compared with child ADHD [Cortese et al., 2012; Hart et al., 2013]. Despite clear evidence for right side dominance in stopping as indicated by the conjunction analyses, we also noted left IFC involvement specifically in stopping at more liberal thresholds, with preliminary evidence of reduced activation in the ADHD group. However, Left IFC involvement in stopping has been noted in meta‐analyses and recent studies [Boehler et al., 2010; Swick et al., 2008, 2011], with reduced activation observed in adult ADHD patients [Cubillo et al., 2010] consistent with present findings.

Despite group differences in the right IFC associated with stopping compared with shifting, activation in this area was noted in both groups separately. Similarly, conjunction analyses indicated ADHD patients appeared to engage broadly the same brain regions as controls whilst performing the task and in particular frontoparietal areas, albeit with reduced extent, a finding common in patient groups. Thus, there were no extensive group differences in frontoparietal areas during stopping and shifting. This is consistent with findings suggesting more subtle and relatively focal hypoactive regions mostly within the frontoparietal network in adult ADHD [Cortese et al., 2012], and with numerous studies failing to find pervasive hypoactivation in adults with ADHD. Nevertheless, despite engaging frontoparietal regions, behavioral impairments and reduced activation in specific control regions were found in the patients. This pattern appears to differ from abnormal brain activations in children with ADHD [Cortese et al., 2012; Hart et al., 2013] and reemphasizes the importance of examining brain function in adults to better understand the adult disorder.

We did not find much evidence for attentional shifting abnormalities in ADHD in behavior or BOLD signal. Few studies have examined shifting or cognitive flexibility in adults, with two studies using traditional task switching paradigms reporting inconsistent results, possibly due to different comorbidity and medication status [Cubillo et al., 2010; Dibbets et al., 2010]. It is difficult to interpret the present findings within the context of these studies as task requirements were very different, with infrequent shifting requiring no change in overt responding [Dodds et al., 2011]. In any case, as evidenced by the conjunction analysis both stopping and shifting recruit adaptive online control activating frontoparietal regions in both groups.

The present study included two go no‐go tasks, with one requiring additional working memory and attentional demands (i.e., the complex stopping task). Although preliminary, the interaction analysis provides tentative evidence supporting the importance of task demands on observed group differences. Some previous studies on executive function have shown that posterior areas of the brain, including the cerebellum, occipital and parietal lobe, and especially the precuneus may show hyperactivation accompanying adequate or impaired performance in ADHD [Dillo et al., 2010; Epstein et al., 2007]. This is especially characteristic of adults and has been interpreted as evidence for compensatory activity [Dibbets et al., 2009]. Current results suggest that with the simple task, as compared with the more complex task, this is indeed the case.

Whilst there was sufficient power to detect task related activations in the patient group, it cannot be excluded that with increased power, additional group differences would be detected. It may also be that functional abnormalities in the ADHD group were mitigated by higher‐than‐average IQ, though the groups were matched and the ADHD group still performed significantly worse. Moreover, this group was clearly clinically impaired, meeting DSM criteria and showing difficulties in daily life due to their ADHD symptoms. It is also possible that, with greater task demands on response control, as in the stop signal or double press tasks, more wide‐spread group differences would have been observed [Cubillo et al., 2010; Dodds et al., 2011; Sebastian et al., 2012]. Moreover, the paradigm did not include control events for the go trials. As go trials involved the presentation of bivalent stimuli, patients may have had difficulties processing them that were not noted in the existing contrasts, although this was not the main focus of the study. Additionally, whilst matched on gender, both male and female subjects were included in our sample. Though the effect sizes in the right IFC suggest a similar pattern of results across gender in the present case, potential gender differences in adult ADHD remain an important avenue for future research [Valera et al., 2010]. Not all patients had a confirmed diagnosis from childhood, though all together with an independent rater reported childhood ADHD‐related problems. In several cases a parent specifically stated that they avoided a childhood diagnosis so as not to stigmatize their child. This stresses some of the difficulties in detection and treatment of some ADHD patients, particularly females. Just over half the patients were taking psychostimulants and although medication was discontinued 24 h before testing, it is also possible that longer‐term effects of the stimulants influenced present results. Some evidence suggests long‐term stimulant use may lead to normalization of some functional abnormalities, albeit most likely in subcortical structures [Hart et al., 2013]. Whilst caution is advised in interpreting medication effects in small samples, we note consistency between the present study and previous ones employing unmedicated patients [Cubillo et al., 2010; Hart et al., 2013].

## CONCLUSION

The results indicate right IFC hypo activation in a group of adult ADHD patients with no comorbidities that was specifically associated with stopping. The use of a highly specific contrast rules out group differences in attentional orienting, arousal and motivation that were likely confounded in many of the previous studies. The results support impaired response control as a core deficit in adult ADHD with associated abnormal functional activation in the IFC. They also demonstrate a source of variability in posterior regions of the brain, which is of interest as different studies employ different versions of response inhibition tasks, varying widely in task demands such as working memory. It is important that future studies examining PFC function in ADHD employ additional control conditions to investigate other response control and executive function abnormalities.
